# Remote Sensing Data to Detect Hessian Fly Infestation in Commercial Wheat Fields

**DOI:** 10.1038/s41598-019-42620-0

**Published:** 2019-04-16

**Authors:** Ganesh P. Bhattarai, Ryan B. Schmid, Brian P. McCornack

**Affiliations:** 1 Department of Entomology, Kansas State University, Manhattan, KS 66506 USA; 2 Plant Biosecurity Cooperative Research Centre, LPO Box 5012, ACT AU 2617 Bruce, Australia; 3 Present Address: Ecdysis Foundation, Estelline, SD 57234 USA

## Abstract

Remote sensing data that are efficiently used in ecological research and management are seldom used to study insect pest infestations in agricultural ecosystems. Here, we used multispectral satellite and aircraft data to evaluate the relationship between normalized difference vegetation index (NDVI) and Hessian fly (*Mayetiola destructor*) infestation in commercial winter wheat (*Triticum aestivum*) fields in Kansas, USA. We used visible and near-infrared data from each aerial platform to develop a series of NDVI maps for multiple fields for most of the winter wheat growing season. Hessian fly infestation in each field was surveyed in a uniform grid of multiple sampling points. For both satellite and aircraft data, NDVI decreased with increasing pest infestation. Despite the coarse resolution, NDVI from satellite data performed substantially better in explaining pest infestation in the fields than NDVI from high-resolution aircraft data. These results indicate that remote sensing data can be used to assess the areas of poor growth and health of wheat plants due to Hessian fly infestation. Our study suggests that remotely sensed data, including those from satellites orbiting >700 km from the surface of Earth, can offer valuable information on the occurrence and severity of pest infestations in agricultural areas.

## Introduction

Remote sensing approaches have been widely used in ecological and agricultural research and management^1,2^. Spectral data collected by remote sensors mounted on aerial platforms such as satellites, manned aircrafts, and small unmanned aircraft systems (sUAS) are used in a broad range of ecological studies such as landcover and vegetation analyses, plant community structure and diversity, and ecosystem functioning^3–6^. However, these data are rarely used to detect insect pests in agricultural areas. Remotely sensed data that have shown promising results in assessing plant chemistry and functional traits in complex ecosystems^6–9^ may also provide critical information on the status and severity of pest infestations in crop fields.

Multi- and hyper-spectral data from a wide range of aerial and handheld sensors are generally used to develop a series of vegetation indices that are related to plant status, chemistry, and physiological activity^10,11^. Normalized difference vegetation index (NDVI) is one of the most common and well understood vegetation indices^1,12^. It is estimated as the normalized difference between leaf reflectance in near-infrared (NIR) region of the spectrum, which is scattered by the mesophyll structures of leaves, and red region that is absorbed by chlorophyll^13,14^. As a reliable predictor of plant health, primary productivity, and standing biomass^1,12^, NDVI serves as an important tool in ecological studies. For example, NDVI derived from satellite data are historically used to map land use classes in terrestrial ecosystems and assess land cover changes in response to anthropogenic and natural disturbances^3,4^. Similarly, it has been used to predict species richness, animal distribution, and animal performance in heterogeneous environments^5,15–17^. NDVI data are also used to evaluate forest defoliation and tree mortality caused by insect outbreaks^18,19^. Moreover, NDVI is a reliable indicator of biotic and abiotic plant stresses that often reduce chlorophyll concentration and, consequently, increase reflectance in red region of the spectrum^20^.

Despite the technological and analytical advances, and associated ecological applications, remotely sensed data are underutilized in the study and management of arthropod pests in agricultural ecosystems. Efforts on this subject are primarily limited to laboratory and field experiments evaluating reflectance spectra of pest infested plants collected by a sensor located within a few meters of the plant canopy. For a variety of crop species (e.g., wheat, soybean, and cotton) and their herbivores (e.g., aphids, and mites), infested plants showed substantially different values for the raw reflectance and indices, primarily NDVI, compared to uninfested plants^21–29^. Pest infested plants consistently showed lower NDVI than the uninfested plants. Other studies that used aircrafts to collect data from a higher elevation documented similar results^30,31^. Recently, hyperspectral data collected from sUAS have been used to map the distribution of cryptic pest species such as grape phylloxera in vineyards^32^. Spectral data from distant platforms, including satellites, have potential to provide frequent, efficient, and cost-effective tools to map pest infestations and spread in agricultural areas.

We performed a field study to evaluate the spectral reflectance of winter wheat (*Triticum aestivum*, Poaceae) fields infested by an introduced insect pest, the Hessian fly (*Mayetiola destructor* (Say); Diptera: Cecidomyiidae). The Hessian fly begins infesting wheat seedlings early in the growing season and results in reduced plant growth, biomass, and grain production^33,34^. In spring 2017, we quantified Hessian fly infestation at multiple sampling points in seven commercial wheat fields in Kansas (KS), USA. Then, we analyzed a series of multispectral satellite and aircraft data to estimate NDVI for each sampling point across the fields. As an indicator of plant health and biomass, NDVI was expected to decrease with pest infestation. Furthermore, we expected high-resolution NDVI from aircraft data (*see* Methods: Remote sensing data) to exhibit a stronger relationship with pest infestation than low-resolution NDVI acquired from satellite data. We also predicted a stronger relationship between NDVI and pest infestation when smaller plot size was used to estimate NDVI for the same sampling points. Using NDVI from different platforms and Hessian fly infestation in the field, we tested the following hypotheses. (1) NDVI will decrease with increased infestation by Hessian fly. (2) The relationship between the pest infestation and NDVI will increase in strength overtime. (3) High-resolution NDVI from aircraft data will exhibit a stronger relationship with pest infestation in the field than low-resolution NDVI from either platform.

## Methods

### Study system

Wheat is one of the major agricultural commodities of the USA. Representing 8.6% of global wheat production between 2007 and 2016, US farmers annually produced 59 million metric tons of wheat planted on >23 million ha of agricultural land^35^. A diverse assemblage of arthropods causes substantial damage to wheat production. Some of the most important pests in the USA include Hessian fly, and several species of aphids, mites, and Lepidopterans^36^. Detecting pests in the field, preferably early in the infestation stages, could guide efficient control measures at targeted areas and minimize loss in productivity. However, vast acreage of wheat fields in the USA imposes severe limitations in employing traditional ground-based surveys to detect insect pests in the field.

The Hessian fly is one of the major insect pests in wheat producing areas around the world^33^. Native to the Fertile Crescent region in the Middle East, Hessian fly is one of the earliest recorded introduced invasive species in North America^37–39^. Hessian fly infestation begins early in the growing season of wheat^33^. In the case of winter wheat in KS, seedlings are infested in fall. Females lay eggs on the upper side of leaves. Once hatched, larvae move towards the base of the plants and establish feeding sites. Larval damage induces nutritive tissue at the base of the leaf that also acts as a nutrient sink within the plant^40^. In the northern states of the US, the insects overwinter as pupae on the plants. Spring infestation may occur following the emergence of adults^41^. Feeding damage to the plant by immature larvae results in slower plant growth, reduced biomass, and occasional death of seedlings causing up to 10% reduction in grain production^33,41,42^. Despite the cost, no effective method has been developed to predict Hessian fly infestation in agricultural landscapes leaving field survey as the only tool to guide pest management^43^. An efficient remote sensing method to map pest prevalence and severity in the field could contribute substantially in the management of this pest.

### Field survey

We surveyed seven commercial winter wheat fields located in Marion and Dickinson Counties, KS (Table 1) to examine whether NDVI estimated from remotely sensed data can detect plant responses related to Hessian fly infestation. Wheat fields differed from each other in various ways including area, topography, previous crops, wheat variety, plant resistance level against key pests, and management practices (Table 1). Fields ranged from ~6 to 32 (mean ± se = 23.7 ± 3.5) ha in area. Wheat planting in all fields was complete by mid October 2016. All fields were planted with moderate to highly susceptible varieties of wheat. In three fields, wheat was planted in the previous year and volunteer wheat between the cropping seasons was controlled. In the other four fields, maize was planted in the previous year, which required no volunteer wheat control. None of these local factors were shown to influence the severity of pest infestation in the fields^43^.

**Table 1:** Commercial winter fields used in the study.

Field ID	County	Coordinate	Wheat variety	Hessian fly resistance level	Volunteer wheat controlled	Crop in previous year	Area (Ha)	Sampling points (N)	Imagery dates	
									Sentinel-2	TerrAvion
1	Marion	38.2107°, −97.1362°	Everest	5	No	Maize	29.53	23	11/01/2016; 01/30/2017; 03/01/2017; 04/10/2017	04/19/2017; 05/05/2017
2	Marion	38.2058°, −97.1365°	LCS Mint	9	No	Maize	18.20	15		
3	Marion	38.1909°, −97.1209°	Everest	5	No	Maize	31.22	50		
4	Marion	38.2159°, −97.1686°	Everest	5	No	Maize	21.65	47		
5	Marion	38.2793°, −97.0886°	T158	9	Yes	Wheat	31.62	50		
6	Dickinson	38.9965°, −97.3566°	WB 4458 & Armour	9	Yes	Wheat	27.81	39		Not available
7	Dickinson	38.9998°, −97.3549°	WB 4458 & Armour	9	Yes	Wheat	6.07	34		Not available

Resistance level of wheat varieties against Hessian fly infestation is reported on a 0 (highly resistant) to 9 (highly susceptible) point scale. A blend of two susceptible varieties were planted in the fields located in Dickinson County.

On 19–22 March 2017, we visited all wheat fields to survey Hessian fly infestations. A uniform grid of sampling points (n = 15 to 50 per site, mean ± se = 37.0 ± 5.2, Table 1) was developed for each site using ArcGIS^®^ 10.2 (ESRI, Redlands, CA). Sampling points within a field were located at least 25 m from each other such that no two adjacent points could share the same pixel in aerial data (*see* Methods: Remote sensing data). Sampling points in each field were located using a Trimble^®^ Recon^TM^ GPS System (Trimble, Dayton, OH). At each sampling point, plants were visually inspected for infestation by other pest species. No sign of noteworthy infestation was observed during field surveys and follow up field visits until the end of April. All wheat plants growing in a 1-m row at each sampling point were collected. After all fields were sampled, each plant from individual sampling point was examined carefully for the presence of Hessian fly puparia by removing leaf sheaths to the base of the stem and the number of puparia was enumerated (*see* Schmid *et al*.^43^ for the detailed methods). These data were used to determine the total number of plants, the proportion of plants infested by Hessian fly, and the average number of puparia per infested plant at each sampling point. Finally, Hessian fly infestation level at each sampling point was estimated as the product of proportion of plants infested and the average number of puparia per infested plant (inverse hyperbolic sine transformed [=ln [x_i_ + (x_i_^2^ + 1)^0.5^]^44^). These data represented the intensity of fall infestation by Hessian fly in the fields and, therefore, predicted to explain the variation on NDVI in the course of this study. Total number of plants along the 1-m row was used as an estimate of plant density at each sampling point.

### Remote sensing data

Multispectral remote sensing data were acquired from two imaging platforms for all wheat fields. First, Level 1 C Sentinel-2 satellite data (S2A-L1C) were acquired for all seven sites. Sentinel-2 is an Earth observation mission developed by the European Space Agency (https://www.esa.int) to monitor terrestrial and coastal ecosystems. Orbiting at 786 km above the Earth’s surface, Sentinel-2 satellites collect optical data on 13 spectral bands in visible, NIR and shortwave infra-red regions. These data are available at 10 m spatial resolution for visible (blue: band center [λ] = 492 nm, width = 98 nm; green: λ = 560 nm, width = 45 nm; red: λ = 665 nm, width = 38 nm) and NIR (λ = 833 nm, width = 145 nm) bands. Other bands that are available at coarser spatial resolution of 20 or 60 m were not suitable for this study. Although each satellite provides global data every 10 days, sensor perspective and atmospheric factors including cloud cover over sites limited the frequency of data availability for our fields to four imagery dates (2016 November 1, 2017 January 30, 2017 March 1, and 2017 April 10). However, those four dates covered most of the growing season of winter wheat in KS. No atmospheric or radiometric correction was applied to these data.

Second, aircraft data for all five sites located in Marion County were acquired from TerrAvion (https://www.terravion.com; San Leandro, CA), a commercial data provider. TerrAvion provides spectral data on visible and NIR bands, and an estimate of plant vigor (NDVI) collected from a manned aircraft flying ~2 km above ground level. NDVI was estimated by the data provider using red (λ = 614 nm, width = 76 nm) and NIR (λ = 855 nm, width = 50 nm) bands. These data are available at high spatial resolution of 17–20 cm per pixel. TerrAvion data were available for only two imagery dates (2017 April 19 and 2017 May 5) in late growing season.

Using NIR and red bands in ArcGIS^®^ 10.2, we developed NDVI maps for each aerial platform and imagery date for all field sites. Then, we extracted mean NDVI values for 100 m^2^ square plots around the sampling points from a series of NDVI maps for each platform. The plot size of 100 m^2^ is equivalent to the area of one pixel for Sentinel-2 satellite data. In contrary, higher spatial resolution of TerrAvion data allowed the extraction of mean NDVI values at a smaller scale of 1 m^2^ around the sampling points. NDVI datasets extracted for different plot sizes were used to evaluate whether high spatial resolution aircraft data at a finer scale exhibited a stronger relationship with pest infestation than those estimated at a coarser scale for either platform.

### Data analysis

We used a separate mixed effect model for each remote sensing platform (Sentinel-2, TerrAvion) and plot size (100 m^2^, 1 m^2^) to examine the relationship between NDVI and Hessian fly infestation in the wheat fields. Hessian fly infestation, imagery date, and infestation × date interactions were treated as fixed effects. To account for the non-independence of sampling points within a site and repeated estimations of NDVI on those points, we treated sampling points nested in the field as random effects. In the case of significant pest infestation × imagery date interaction, separate mixed effect model was run for each imagery date. Quantile-quantile plots were used to evaluate residual distribution and detect the observations with undue influence for each statistical model. In the cases of outliers, the statistical model was re-run excluding the outlier and the outcomes were evaluated. Finally, goodness of fit of each mixed model is reported as marginal (*R*^2^_*m*_, variance explained by fixed effects) and conditional *R*^2^ (*R*^2^_*c*_, variance explained by the entire model)^45^.

We also evaluated the effect of plant density on NDVI from different platforms and plot sizes. A mixed effect model was developed for each set of NDVI data using plant density, imagery date, and plant density × imagery date interaction as fixed effects and field as a random effect. Similarly, we analyzed the effect of Hessian fly infestation on plant density.

Finally, we used model selection procedure to identify the most informative set of NDVI data in explaining Hessian fly infestation in the fields^46^. Fields in Dickinson County (n = 2) were excluded in model selection because TerrAvion data were not available for those fields. A set of NDVI data that was closest in time to field surveys for Hessian fly infestation was selected for each aerial platform (Sentinel-2: 10 April 2017; TerrAvion: 19 April 2017). In this way, three sets of NDVI data (i.e., determined at 100 m^2^ plots for each platform, and at 1 m^2^ plots for TerrAvion) were used to develop a series of candidate models. Each candidate model was developed as a mixed effect model evaluating the effect of each set of NDVI, a fixed effect, on pest infestation. Field was included in each model as a random effect. Then, we used Akaike information criteria corrected for finite sample size (AIC_c_) to identify the best model explaining the pest infestation in the fields^46^. Candidate models with ΔAIC_c_ > 2 were deemed significantly less informative than the best model. All statistical analyses were performed in R 3.5.0^47^ using ‘nlme’^48^ and ‘MuMIn’^49^ libraries.

## Results

**Figure 1:**
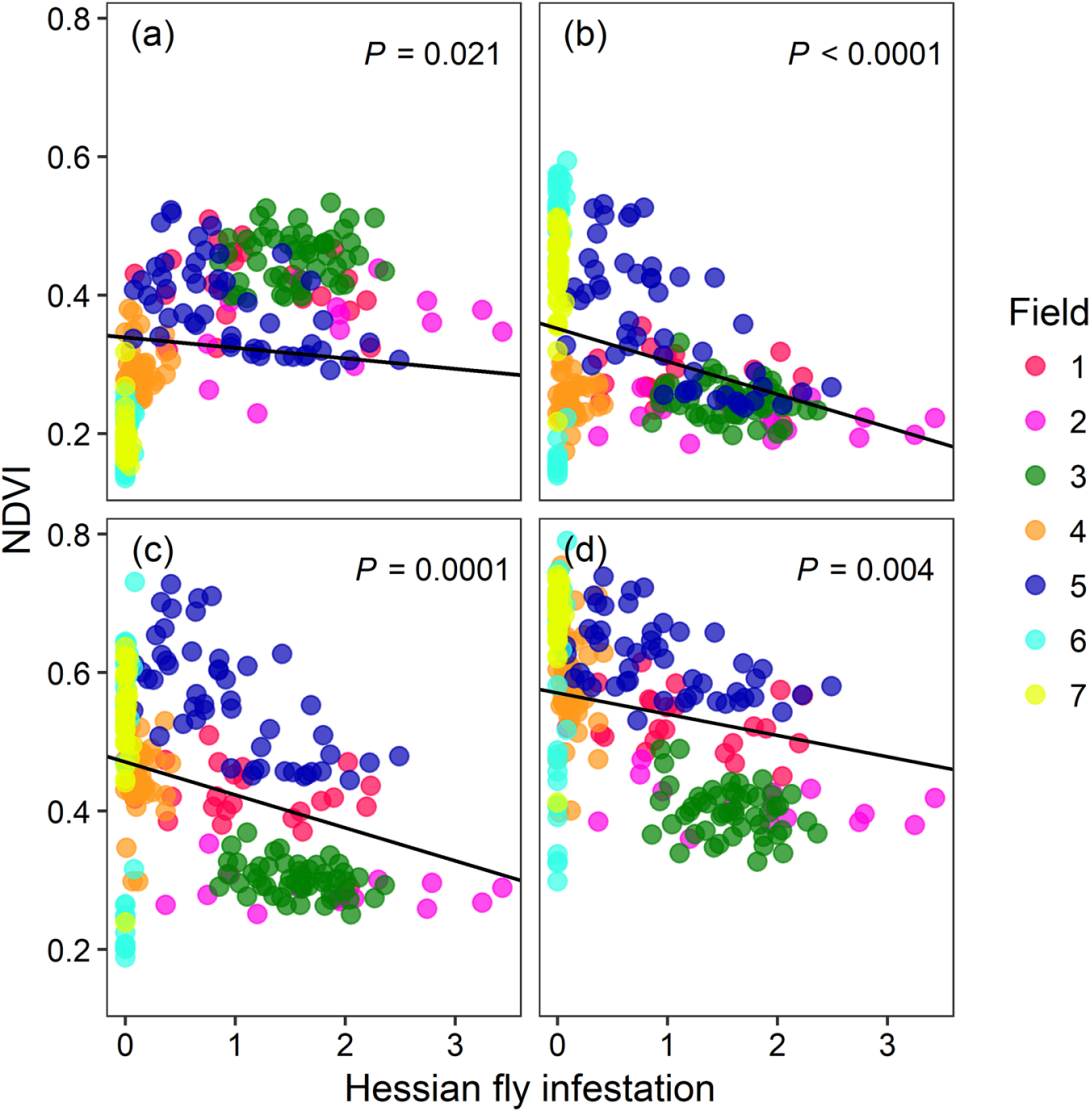
Normalized difference vegetation index (NDVI) determined from Sentinel-2 satellite data decreased with Hessian fly infestation in the commercial wheat fields in KS, USA. Hessian fly infestation at each sampling point was determined as the product of proportion of stems infested and inverse hyperbolic sine transformed number of puparia per stem. Relationship between NDVI (calculated at 100 m^2^ plots) and the level of pest infestation are shown for each imagery date: (**a**) 2016-11-01, (**b**) 2017-01-30, (**c**) 2017-03-01, and (**d**) 2017-04-10. The line fit for each date represents the relationship between NDVI and pest infestation determined from a linear mixed effect model.

**Figure 2:**
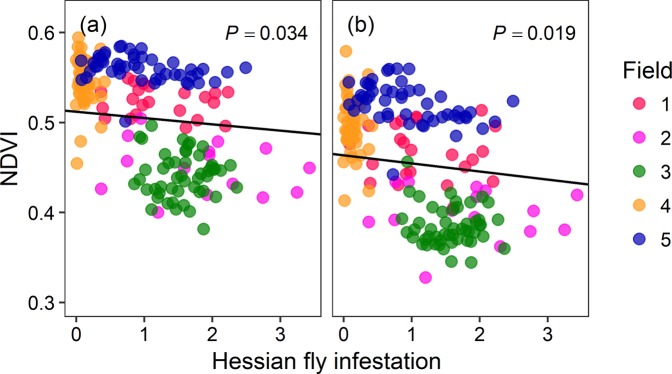
Normalized difference vegetation index (NDVI) determined from TerrAvion aircraft data decreased with Hessian fly infestation in the commercial wheat fields in KS, USA. Hessian fly infestation at each sampling point was determined as the product of proportion of stems infested and inverse hyperbolic sine transformed number of puparia per stem. Relationship between NDVI (calculated at 100 m^2^ plots) and the level of pest infestation are shown for each imagery date: (**a**) 2017-04-19, and (**b**) 2017-05-05. The line fit for each date represents the relationship between NDVI and pest infestation determined from a linear mixed effect model.

NDVI estimated from each remote sensing platform declined with increasing Hessian fly infestation in the fields (Figs 1, 2). For Sentinel-2 satellite data, NDVI increased with time (*F*_3, 768_ = 612.71, *P* < 0.0001) but decreased with pest infestation (*F*_1, 250_ = 14.97, *P* < 0.0001, *R*^2^_*m*_ = 0.58, *R*^2^_*c*_ = 0.78, Fig. 1). A weak, but statistically significant, negative effect of pest infestation on NDVI was evident in November 2016 (*P* = 0.021) that strengthened in intensity in the subsequent imagery dates in 2017 (January 30: *P* < 0.0001; March 1: *P* < 0.0001; April 10: *P* = 0.004; Fig. 1). Although the relationship between NDVI and infestation level was always negative, substantial change in the intensity of this relationship over time was indicated by a significant infestation × date interaction (*F*_3, 768_ = 222.93, *P* < 0.0001). On average, NDVI increased by ~67% across the fields from November 2016 to April 2017.

Consistent with the patterns observed in Sentinel-2 data, NDVI estimated from TerrAvion data was negatively related to Hessian fly infestation (*F*_1, 179_ = 5.59, *P* = 0.019, *R*^2^_*m*_ = 0.19, *R*^2^_*c*_ = 0.96, Fig. 2). NDVI decreased by 10% from April 19 to May 5 (*F*_1, 183_ = 1628.22, *P* < 0.0001). Evaluating these relationships using NDVI data extracted for 1 m^2^ plots around the sampling points caused no noticeable change in the results. NDVI determined at the finer plot size was also negatively related to Hessian fly infestation (*F*_1, 179_ = 4.77, *P* = 0.030, *R*^2^_*m*_ = 0.18, *R*^2^_*c*_ = 0.89).

**Figure 3:**
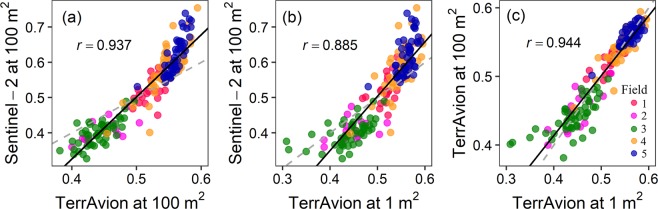
Relationship between normalized difference vegetation index (NDVI) determined from Sentinel-2 and TerrAvion platforms and estimated for the sampling points at different plot sizes. Correlation between NDVI data from (**a**) Sentinel-2 and TerrAvion at 100 m^2^ plots; (**b**) Sentinel-2 at 100 m^2^ and TerrAvion at 1 m^2^ plots; and (**c**) TerrAvion at 100 m^2^ and 1 m^2^ plots. All correlation coefficients are significant (*P* < 0.0001). Solid line represents the line fit between NDVI data sets using least-square regression, and dashed line represents 1:1 relationship between them.

There was a strong correlation between NDVI values derived from Sentinel-2 and TerrAvion platforms at the same sized plots of 100 m^2^ (*r* = 0.937, *P* < 0.0001, Fig. 3a), and at 100 m^2^ plots for Sentinel-2 and 1 m^2^ for TerrAvion (*r* = 0.885, *P* < 0.0001, Fig. 3b). For TerrAvion platform, NDVI data extracted at different sized plots were strongly correlated (*r* = 0.944, P < 0.0001, Fig. 3c). However, NDVI for none of the platforms was related to plant density (Sentinel-2: *P* = 0.40; TerrAvion at 100 m^2^: *P* = 0.46; TerrAvion at 1 m^2^: *P* = 0.26). Furthermore, there was no significant relationship between plant density and Hessian fly infestation (*P* = 0.65) in the fields.

Despite low resolution, NDVI derived from Sentinel-2 satellite data was the best predictor of Hessian fly infestation in the field (AIC_c_ = 286.4, AIC_c_ weight = 0.986). For TerrAvion, there was no substantial difference in AIC_c_ between the models with NDVI estimated at 100 m^2^ (AIC_c_ = 295.8, AIC_c_ weight = 0.009) and 1 m^2^ (AIC_c_ = 296.9, AIC_c_ weight = 0.005) plots.

## Discussion

This study provides evidence that changes in wheat fields in response to insect infestation can be detected by optical sensors mounted in aerial platforms including satellites orbiting >700 km above the Earth surface. These results are consistent with our hypothesis that NDVI, an indicator of plant biomass and health, declines with the increasing Hessian fly infestation. Areas associated with higher levels of pest infestation showed lower NDVI values compared to those with lower levels of pest infestation. These results are most likely driven by reduced biomass and/or poor health of plants growing in the areas of greater pest infestation. Spectral data collected from an aircraft flying ~2 km above the ground provided support to these patterns. Interestingly, neither pest infestation nor NDVI from either platform was related to plant density. These results suggest that the relationships between NDVI and Hessian fly infestation are primarily governed by plant biomass or other plant traits related to herbivory rather than plant density. Furthermore, this study highlights the significance of low-resolution satellite data in mapping and managing pest infested areas in crop fields.

Negative relationships between pest infestation and plant performance are well documented in Hessian fly-wheat interactions^33,34,41,42^. After finding a suitable place in the leaf sheath of a seedling, newly emerged Hessian fly larvae induce gall formation that provides the immature stages with protective feeding sites potentially serving as a nutrient sink in the plant^40^. Damage by larvae could result in low NDVI of an individual plant and, consequently, the infested area in several ways. First, larval feeding substantially reduces plant growth for the entire growing season and can occasionally cause plant death^33,41^. For the plants that showed stem elongation after larval feeding, stem lodging and death may occur^34^. These processes result in smaller, damaged, and unhealthy plants. Second, various plant species under a biotic or an abiotic stress show increased reflectance in the red region of light spectrum, an indication of low chlorophyll concentration^20^. Third, death of tillers and seedlings due to pest infestation may create sparse or empty patches in the areas of severe infestation. All these mechanisms contribute to lower NDVI of the individual plant and the areas under severe infestation. Although the density of seedlings at the time of field survey was not related to pest infestation, decreased plant density at the later time in response to insect damage was possible. Conversely, Hessian fly infested plants are reported to appear darker blue-green in color during early stages of plant growth^34,36^. However, analysis of individual blue and green spectral bands did not exhibit such patterns (GP Bhattarai unpublished data). Therefore, the negative relationship between Hessian fly infestation and NDVI can be attributed to the reduced biomass and/or poor health of the plants. Reduced NDVI of plants in response to the severity of herbivore damage likely represents a general pattern in plant-herbivore interactions. We assert that these approaches can provide valuable information on pest infestation and dynamics for a variety of plant species in agricultural and natural areas.

Analysis of spectral data in relation to pest infestation in crop plants are primarily limited to NDVI and a few additional vegetation indices. For example, NDVI differed substantially between control and pest infested plants for cotton^26,27^, soybean^29^, and wheat^21–25,28^. All these studies involved piercing-sucking and rasping insect pests, aphids and mites, respectively. Using reflectance data collected from a close distance to the plant canopy, these studies provide unequivocal evidence of lower NDVI for crop species suffering from herbivory. Other studies that used similar data collected by sensors mounted on aircrafts revealed the same patterns for aphid infested cotton and wheat plants^30,31^. Although our data showed similar patterns, underlying mechanisms for the negative relationship between pest infestation and NDVI may differ between piercing-sucking or rasping and galling insects. For plants infested by piercing-sucking insects, lower NDVI could be the outcome of leaf chlorosis, reduced moisture content, and reduced biomass^12,20^. In the case of Hessian fly, such patterns most likely result from lower biomass of the infested plants or plant death associated with pest infestation. Hessian fly-induced plant mortality was not measured in the current study, but should be a focus of future research.

Although spectral data captured by sensors from a close distance to the plant canopy have been shown to distinguish between infested and uninfested plants, these approaches require improvement to cover a larger spatial scale and have practical use in pest management. Using multispectral data from higher elevation platforms, we elaborate these approaches to make pest detection and monitoring feasible at larger spatial and temporal scales. We also document that relatively low resolution (100 m^2^ per pixel) orbital data, that were strongly correlated with high resolution aircraft data, efficiently represent the variability in plant performance across the fields. Such variations detected from orbital data and validated through field surveys can play an important role in pest management. On the other hand, recently developed imaging spectroscopy methods are shown to provide detailed assessment of several morphological and chemical traits related to nutritional, functional, and defense status of plants^7–9,50^. These methods exhibit tremendous potential to revolutionize survey and monitoring approaches in relatively homogeneous agricultural landscapes.

We discovered a substantial heterogeneity on the prevalence and severity of Hessian fly infestation within and among wheat fields (*see* also Schmid *et al*.^43^). Hessian fly infestation showed patchy distribution within a field^43^. Two of the field sites located in Dickinson County suffered from nearly non-existent pest infestation. Occurrence and severity of Hessian fly infestation were independent of the local and landscape level factors^43^, which highlights the challenges in field monitoring using ground-based surveys. Out of several factors evaluated by Schmid *et al*.^43^, proportion of wheat cover around the field in the previous year was the only factor that was related to pest infestation. Interestingly, greater coverage of landscape by wheat fields was associated with the lower infestation in the following year. Such a paucity of data on the relevance of local and landscape factors in driving Hessian fly infestation leaves the managers and researchers with the only option of regular field surveys and monitoring. Current practices are inefficient when pest pressure is low to non-existent and impractical when host cover is measured in millions of hectares, which is the case for wheat in KS. In this context, development of a reliable remote sensing method using low-cost satellite and aircraft data will play an important role in pest management.

In this study, we demonstrate the relevance of commonly used vegetation indices estimated from remotely sensed data in mapping insect pest infestation and plant performance in agricultural areas. These findings highlight the opportunity for applying readily available orbital data to map agricultural areas under various biotic or abiotic stresses enabling the managers to make an immediate evaluation and response to mitigate the problem. Our study further indicates that low resolution satellite data could provide more accurate information regarding spatial distribution and severity of pest infestation in an agricultural landscape. On the other hand, these methods and findings open the possibility of hierarchical studies involving multiple aerial platforms, including satellites, aircrafts (both manned and unmanned), and ground observations, to study plant-insect interactions at the larger temporal and spatial scales. Those studies could also help understating the contribution of local (e.g., host quality, competition, enemy pressure, management practices, etc.) and large scale (e.g., climate, biogeography, etc.) factors on the spread and dynamics of insect pests.

## Data Availability

Data are available from the corresponding author upon reasonable request.

## References

[1] Kerr JT, Ostrovsky M (2003). From space to species: ecological applications for remote sensing. Trends Ecol. Evol..

[2] Nansen C, Elliott N (2016). Remote sensing and reflectance profiling in Entomology. Annu. Rev. Entomol..

[3] Tucker CJ, Townshend JRG, Goff TE (1985). African land-cover classification using satellite data. Science.

[4] Hansen MC, Defries RS, Townshend JRG, Sohlberg R (2000). Global land cover classification at 1 km spatial resolution using a classification tree approach. Int. J. Remote Sens..

[5] Hurlbert AH, Haskell JP (2003). The effect of energy and seasonality on avian species richness and community composition. Am. Nat..

[6] Jetz W (2016). Monitoring plant functional diversity from space. Nat. Plants.

[7] Asner GP, Martin RE, Anderson CB, Knapp DE (2015). Quantifying forest canopy traits: imaging spectroscopy versus field survey. Remote Sens. Environ..

[8] Schneider FD (2017). Mapping functional diversity from remotely sensed morphological and physiological forest traits. Nat. Commun..

[9] Martin RE (2018). An approach for foliar trait retrieval from airborne imaging spectroscopy of tropical forests. Remote Sens..

[10] Haboudane D, Miller JR, Tremblay N, Zarco-Tejada PJ, Dextraze L (2002). Integrated narrow-band vegetation indices for prediction of crop chlorophyll content for application to precision agriculture. Remote Sens. Environ..

[11] Thenkabail, P. S., Lyon, J. G. & Huete, A. Advances in hyperspectral remote sensing of vegetation and agricultural croplands. [Thenkabail, P. S., Lyon, J. G. & Huete, A. (eds)] *Hyperspectral Remote Sensing of Vegetation*. 3–35 (CRC press, 2012).

[12] Pettorelli N (2005). Using the satellite-derived NDVI to assess ecological responses to environmental change. Trends Ecol. Evol..

[13] Sinclair TR, Hoffer RM, Schreiber MM (1971). Reflectance and internal structure of leaves from several crops during a growing season. Agron. J..

[14] Rouse, J. W., Haas, R. H., Schell, J. A., Deering, D. W. & Harlan, J. C. Monitoring the vernal advancements and retrogradation of natural vegetation (NASA/GSFC, Greenbelt, MD, USA, 1973).

[15] Gould W (2000). Remote sensing of vegetation, plant species richness, and regional biodiversity hotspots. Ecol. Appl..

[16] Nagendra H, Gadgil M (1999). Biodiversity assessment at multiple scales: Linking remotely sensed data with field information. P. Natl. Acad. Sci. USA.

[17] Fairbanks DHK, McGwire KC (2004). Patterns of floristic richness in vegetation communities of California: regional scale analysis with multi-temporal NDVI. Global Ecol. Biogeogr..

[18] Eklundh L, Johansson T, Solberg S (2009). Mapping insect defoliation in Scots pine with MODIS time-series data. Remote Sens. Environ..

[19] Spruce JP (2011). Assessment of MODIS NDVI time series data products for detecting forest defoliation by gypsy moth outbreaks. Remote Sens. Environ..

[20] Carter GA, Knapp AK (2001). Leaf optical properties in higher plants: linking spectral characteristics to stress and chlorophyll concentration. Am. J. Bot..

[21] Yang Z, Rao MN, Elliott NC, Kindler SD, Popham TW (2005). Using ground-based multispectral radiometry to detect stress in wheat caused by greenbug (Homoptera: Aphididae) infestation. Comput. Electron. Agr..

[22] Yang Z, Rao MN, Elliott NC, Kindler SD, Popham TW (2009). Differentiating stress induced by greenbugs and Russian wheat aphids in wheat using remote sensing. Comput. Electron. Agr..

[23] Mirik M (2006). Using digital image analysis and spectral reflectance data to quantify damage by greenbug (Hemiptera: Aphididae) in winter wheat. Comput. Electron Agr..

[24] Mirik M, Michels GJ, Kassymzhanova-Mirik S, Elliott NC (2007). Reflectance characteristics of Russian wheat aphid (Hemiptera: Aphididae) stress and abundance in winter wheat. Comput. Electron Agr..

[25] Mirik M, Ansley RJ, Michels GJ, Elliott NC (2012). Spectral vegetation indices selected for quantifying Russian wheat aphid (Diuraphis noxia) feeding damage in wheat (Triticum aestivum L.). Precis. Agr..

[26] Reisig DD, Godfrey LD (2006). Remote sensing for detection of cotton aphid - (Homoptera: Aphididae) and spider mite - (Acari: Tetranychidae) infested cotton in the San Joaquin Valley. Environ. Entomol..

[27] Reisig DD, Godfrey LD (2007). Spectral response of cotton aphid - (Hemoptera: Aphididae) and spider mite - (Acari: Tetranychidae) infested cotton: controlled studies. Environ. Entomol..

[28] Yuan L (2014). Spectral analysis of winter wheat leaves for detection and differentiation of diseases and insects. Field Crop Res..

[29] Alvis TM, Macrae IV, Koch RL (2015). Soybean aphid (Hemiptera: Aphididae) affects soybean spectral reflectance. J. Econ. Entomol..

[30] Elliott N (2007). Airborne multi-spectral remote sensing of Russian wheat aphid injury to wheat. Southwest. Entomol..

[31] Reisig DD, Godfrey LD (2010). Remotely sensing arthropod and nutrient stressed plants: a case study with nitrogen and cotton aphid - (Hemiptera: Aphididae). Environ. Entomol..

[32] Vanegas F, Bratanov D, Powell K, Weiss J, Gonzalez F (2018). A novel methodology for improving plant pest surveillance in vineyards and crops using UAV-based hyperspectral and spatial data. Sensors.

[33] Stuart JJ, Chen MS, Shukle R, Harris MO (2012). Gall midge (Hessian flies) as plant pathogens. Annu. Rev. Phytopathol..

[34] Schmid RB, Knutson AE, Giles KL, McCornack BP (2018). Hessian fly (Diptera: Cecidomyiidae) biology and management in wheat. J. Integr. Pest Manag..

[35] USDA. Wheat data: yearbook tables. United States Department of Agriculture, Economic Research Service https://www.ers.usda.gov/data-products/wheat-data/ (2018).

[36] Whitworth, R. J., Sloderbeck, P. E. & Davis, H. N. *Crop Insects in Kansas* (Kansas State University, Manhattan, KS, USA, 2010).

[37] Howard, L. O. *A History of Applied Entomology* (Smithsonian Institute, City of Washington, USA, 1930).

[38] Elton, C. S. *The Ecology of Invasions by Animals and Plants* (Methuen, London, UK, 1958).

[39] Pauly PJ (2002). Fighting the Hessian fly: American and British responses to insect invasion: 1776–1789. Environ. Hist..

[40] Harris MO (2006). Virulent Hessian fly (Diptera: Cecidomyiidae) larvae induce a nutritive tissue during compatible interactions with wheat. Ann. Entomol. Soc. Am..

[41] Buntin GD (1999). Hessian fly (Diptera: Cecidomyiidae) injury and loss of winter wheat grain yield and quality. J. Econ. Entomol..

[42] Buntin GD, Ott SL, Johnson JW (1992). Integration of plant-resistance, insecticides, and planting date for management of the Hessian fly (Diptera, Cecidomyiidae) in winter-wheat. J. Econ. Entomol..

[43] Schmid RB, Hefley T, Lollato R, McCornack BP (2019). Landscape effects on Hessian fly, *Mayetiola destructor* (Diptera: Cecidomyiidae), distribution within six Kansas commercial wheat fields. Agric. Ecosyst. Environ..

[44] Burbidge JB, Magee L, Robb AL (1988). Alternative transformation to handling extreme values of the dependent variable. J. Am. Stat. Assoc..

[45] Nakagawa S, Schielzeth H (2013). A general and simple method for obtaining R^2^ from generalized linear mixed‐effects models. Methods Ecol. Evol..

[46] Burnham, K. P. & Anderson, D. R. *Model Selection and Multimodel Inference: A Practical Information-Theoretic Approach*, 2nd ed. (Springer, New York, 2002).

[47] R Core Team. R: A language and environment for statistical computing. R Foundation for Statistical Computing, Vienna, Austria, https://www.R-project.org/ (2018).

[48] Pinheiro, J. *et al*. Nlme: Linear and Nonlinear Mixed Effects Models. R package version 3.1-137, https://CRAN.R-project.org/package=nlme (2018).

[49] Barton, K. MuMIn: Multi-Model Inference. R package version 1.40.4, https://CRAN.R-project.org/package=MuMIn (2018).

[50] Asner GP (2017). Airborne laser-guided imaging spectroscopy to map forest trait diversity and guide conservation. Science.

